# Preferences of German and Swiss melanoma patients for toxicities versus melanoma recurrence during adjuvant treatment (GERMELATOX-A-trial)

**DOI:** 10.1007/s00432-023-05027-z

**Published:** 2023-07-05

**Authors:** Katharina C. Kähler, S. Hüning, D. Nashan, F. Meiss, D. A. Rafei-Shamsabadi, H. Rissmann, C. Colapietro, E. Livingstone, L. V. Maul, M. Heppt, J. C. Hassel, R. Gutzmer, C. Loquai, L. Heinzerling, M. M. Sachse, A. S. Bohne, L. Moysig, W. Peters, J. Rusch, C. Blome

**Affiliations:** 1 Department of Dermatology, University Hospital Schleswig-Holstein (UKSH), Campus Kiel, Kiel, Germany; 2 Department of Dermatology, Dortmund, Germany; 3 Department of Dermatology, Medical Center-University of Freiburg, Faculty of Medicine, University of Freiburg, Freiburg, Germany; 4 Department of Dermatology, University Hospital Essen, Essen, Germany; 5 Department of Dermatology, University Hospital Basel, Basel, Switzerland; 6 Department of Dermatology, Uniklinikum Erlangen, Friedrich-Alexander University (FAU) Erlangen-Nürnberg, University Hospital Erlangen, Erlangen, Germany; 7 Comprehensive Cancer Center Erlangen-European Metropolitan Area of Nuremberg (CCC ER-EMN), Erlangen, Germany; 8 Department of Dermatology and National Center for Tumor Therapy (NCT), University Hospital Heidelberg, Heidelberg, Germany; 9 Department of Dermatology, Johannes Wesling Medical Center Minden, Ruhr University Bochum Medical School, Bochum, Germany; 10 Department of Dermatology, Klinikum Bremen-Ost, Gesundheitnord gGmbH, Bremen, Germany; 11 Department of Dermatology and Allergy, University Hospital, LMU Munich, Munich, Germany; 12 Department of Dermatology, Bremerhaven, Germany; 13 Institute for Health Services Research in Dermatology and Nursing (IVDP), University Medical Center Hamburg, Hamburg, Germany

**Keywords:** Patient preferences, Melanoma, Treatment toxicity, Adjuvant treatment

## Abstract

**Purpose:**

Adjuvant treatment with immune checkpoint inhibitors like PD1-antibodies (ICI) ± CTLA4-antibodies (cICI) or targeted therapy with BRAF/MEK inhibitors (TT) in high-risk melanoma patients demonstrate a significant improvement in disease-free survival (DFS). Due to specific side effects, the choice of treatment is very often driven by the risk for toxicity.

This study addressed for the first time in a multicenter setting the attitudes and preferences of melanoma patients for adjuvant treatment with (c)ICI and TT.

**Methods:**

In this study (“GERMELATOX-A”), 136 low-risk melanoma patients from 11 skin cancer centers were asked to rate side effect scenarios typical for each (c)ICI and TT with mild-to-moderate or severe toxicity and melanoma recurrence leading to cancer death. We asked patients about the reduction in melanoma relapse and the survival increase at 5 years they would require to tolerate defined side-effects.

**Results:**

By VAS, patients on average valued melanoma relapse worse than all scenarios of side-effects during treatment with (c)ICI or TT. In case of severe side effects, patients required a 15% higher rate of DFS at 5 years for (c)ICI (80%) compared to TT (65%). For survival, patients required an increase of 5–10% for melanoma survival during (c)ICI (85%/80%) compared to TT (75%).

**Conclusion:**

Our study demonstrated a pronounced variation of patient preferences for toxicity and outcomes and a clear preference for TT. As adjuvant melanoma treatment with (c)ICI and TT will be increasingly implemented in earlier stages, precise knowledge of the patient perspective can be helpful for decision making.

**Supplementary Information:**

The online version contains supplementary material available at 10.1007/s00432-023-05027-z.

## Introduction

Dramatic improvements in melanoma survival have been reported since the advent of targeted therapies and immunotherapies for patients with advanced melanoma (Garutti et al. 2022). Given the success with immunotherapy and targeted therapy in the metastatic setting, there has been a natural progression of this treatment into the adjuvant setting, where we can provide benefit to patients considered to be at high risk for recurrence and death from melanoma. High-risk melanoma is defined as a deep invasive primary tumor with or without ulceration [AJCC (8th edition) stage IIB and IIC] or with regional nodal disease (AJCC stage III). 10-year melanoma-specific survival rates range from 84% for AJCC stage II down to 69% for AJCC stage III (Gershenwald et al. 2017). Therefore, some patients will progress and develop recurrence or metastatic disease, while others can be cured with surgery alone. Hence, it is important to discuss when to use adjuvant therapy, whether we should treat in the adjuvant setting or wait until recurrence, and whether the benefits of adjuvant therapy outweigh the risks. Adjuvant therapies like immune checkpoint blockade or targeted therapy have been approved and are now considered standard of care not only for high-risk patients but also for intermediate risk patients in AJCC stage IIB. These therapies have shown improvements for disease-free survival (DFS) and also distant metastasis survival (DMFS) which is used as surrogate parameter for overall survival (Kobeissi and Tarhini 2022; Long et al. 2022). Adjuvant therapy is considered potentially curative and avoids the morbidity of relapsed disease and the still poor outcomes seen in metastatic disease. In stage IV, adjuvant treatment with (c)ICI has also demonstrated to be very efficacious and is, therefore, increasingly used in the routine (Livingstone et al. 2022).

The toxicity of TT, namely dabrafenib and trametinib, is characterized by symptoms like fever, gastrointestinal complaints, joint pain, a decrease of the left ventricular function, and eye disorders (Lazaroff and Bolotin 2023). In contrast, c(ICI) induces autoimmune side effects in nearly every organ system that could lead in a small subset of patients to a fatal course (Wang et al. 2018). In the majority of patients, health-related quality of life (HrQoL) is not or only temporarily impaired (Bottomley et al. 2021; Khattak et al. 2022; Pedersen et al. 2023). In case of severe side effects, HRQoL may be persistently impaired, which can eventually lead to treatment cessation (Pedersen et al. 2023; Wang et al. 2018).

In contrast to TT, immunotherapy has the potential for substantial toxicity that may be chronic (Schulz et al. 2022) and up to lifelong; hence, discussion of risks and benefits of therapy is of importance.

The balance between the benefits of treatment and the risk of toxicity will ultimately have to be made by our patients. To date, HRQoL has been analyzed in many (pivotal) trials to determine if toxicity during adjuvant treatment alters quality of life. However, available data about patient preferences for benefit versus toxicity in these treatments in the adjuvant setting are limited (Liu et al. 2019; Livingstone et al. 2020). This study will, to our best knowledge, be the first to investigate in a multicenter approach how melanoma patients not biased by a current treatment decision situation value different spectrums of toxicity in the adjuvant setting.

## Methods

### Patients and study centers

Ten German skin cancer centers and one Swiss skin cancer center with high expertise in treating melanoma were involved in this cross-sectional, observational non-interventional questionnaire study.

Patients with low-risk melanoma, defined as T1a, min. 8 weeks after initial diagnosis, no sentinel node biopsy or significant co-morbidities were eligible. Patients without physical or mental capacity to participate or insufficient German language skills were excluded. The rationale for low-risk melanoma patients was to choose a patient cohort with the experience of melanoma diagnosis, but not in the situation of having to decide for or against adjuvant treatment, in order to avoid ethical conflicts potentially induced by this study that may influence a patient’s decision.

We asked for sociodemographic data like age, gender, marital status, employment, and working hours as well as experience with cancer and co-morbidities.

### Treatment trade-off

As no validated survey tool for the objective of our study existed, the questionnaire was developed de novo. Treatment scenarios within the questionnaire were based on the literature and the expertise of two clinical oncologists. The questionnaire was pre-tested for comprehensibility by three independent physicians and four volunteering patients and revised accordingly.

Preferences were elicited with a paper-based treatment-trade-off task. Participants were asked to imagine being in the situation of having a melanoma with a 30% chance of 5-year DFS and a 50% chance of 5-year OS. Treatment preferences were elicited as follows: each of the three treatments (TT; ICI; or cICI treatment) was described, including the respective nature and probability of side effects. An additional scenario evaluated preferences for recurrence of melanoma after adjuvant treatment. This resulted in 12 different scenarios (example in the supplementaries) that were described:

Scenario 1 = TT without side effects.

Scenario 2 = TT with mild to moderate side effects.

Scenario 3 = TT with severe side effects.

Scenario 4 = ICI without side effects.

Scenario 5 = ICI with mild to moderate side effects.

Scenario 6 = ICI with mild-to-moderate side effects and abnormal blood values.

Scenario 7 = ICI with severe side effects.

Scenario 8 = cICI without side effects.

Scenario 9 = cICI with mild-to-moderate side effects.

Scenario 10 = cICI with mild-to-moderate side effects and abnormal blood values.

Scenario 11 = cICI with severe side effects.

Scenario 12 = Recurrence of melanoma after adjuvant treatment (only rated for acceptability).

In contrast to previous uses of treatment-trade-off, participants were not presented a series of different DFS and OS rates for each scenario (Jansen et al. 2001), but were asked to directly state the minimum number of prevented relapses or deaths required for them to choose the treatment over the alternative of not receiving treatment (i.e., the chance of DFS and OS needed for them to choose the treatment versus no treatment). The statement to be completed read, for example, "I would choose the treatment described in scenario 1 if it would prevent a relapse in at least ___ of these 70 patients."

Patients were additionally asked to rate the acceptability of each scenario using visual analog scales (VAS) ranging from 0% = completely unbearable to 100% = completely bearable.

Thus, for each scenario, patients rated the minimally required increase in DFS and OS, respectively, as well as acceptability using the VAS.

### Primary endpoint

The primary endpoint was to determine patient preferences during TT with severe side effects in an adjuvant treatment setting, defined as the minimally required benefit in terms of the additional chance of 5-year DFS, as stated in the treatment trade-off task.

### Additional assessments

We assessed dosage form preferences (infusion vs. oral medication) by asking patients to state their agreement with the following statements on a five-point scale ranging from “completely agree” to “do not agree at all”:

*Self-applied medication*: “It is okay for me to take the medicine on my own”.

*Supervised medication*: “It seems beneficial to me to have the drug administered under the supervision of a doctor”.

*Rather visits than self-application*: “I'm happy to put up with infusions and more frequent visits to the doctor, as long as I then don't have to be responsible for taking the medicine myself”.

*Acceptance of long doctor’s appointments*: “I can accept that an appointment with infusion and medical examination can take several hours”.

*Compliance to a strict intake schedule*: “I can stick to a precise schedule for taking pills”.

Importance of treatment method (infusion vs. pill): “The way I get the medicine administered (infusion or pills) matters to me”.

In addition, patients rated their preference for dosage via infusion vs. pill on a horizontal VAS from − 100 (infusion) to + 100 (pills) and 0 indicating "undecided".

Furthermore, we evaluated depression and anxiety using the Hospital Anxiety and Depression Scale (HADS) (Zigmond and Snaith 1983) ranging from 0 to 21 with higher values indicating higher anxiety or depression, respectively; cancer-specific HRQoL using the EORTC-QLQ-C30 version 3.0 (Bjordal et al. 2000) ranging from 0 to 100 with higher scores indicating better HRQoL; and generic HRQoL using the EQ-5D-5L and EQ VAS (Herdman et al. 2011), ranging from 0 to 1 and 0 to 100, respectively, with higher scores indicating better health.

### Sample size calculation

The number of patients to be included was determined according to the primary endpoint of patients’ preferences for BRAF/MEKi treatment. In order to determine the percentage of patients who would choose BRAF/MEKi treatment at a 5-year-DFS of 65% or lower with a 95% confidence interval width of ± 10 percentage points, 104 analyzable data sets were needed (or less if the distribution of patients would differ from 50:50; calculated with PASS Sample Size 2008).

### Statistical approach

For all variables, descriptive statistics were computed (frequencies, percentages, mean, median, and/or standard deviation (SD), as applicable).

Participants were excluded from the OS, DFS, or VAS analysis, respectively, if they misordered two or more pairs of scenarios (e.g. lower rank for mild-to-moderate side effects than for severe side effects in otherwise identical scenarios) as this was regarded as an indicator of insufficient understanding of the rating task.

OS, DFS, and VAS were was analyzed as the arithmetic mean along with the 95% confidence interval. Differences between treatment scenarios were tested with paired samples *t* tests. Significance levels equal to or below 0.05 were regarded statistically significant; no adjustment for multiple testing was performed.

The association of treatment preferences (DFS, OS, VAS) with respondent characteristics (socio-demographic data, self-experience with cancer, psychological constructs) was assessed using bivariate tests (Pearson correlations or *t* tests, depending on variable scaling).

## Results

**Fig. 1:**
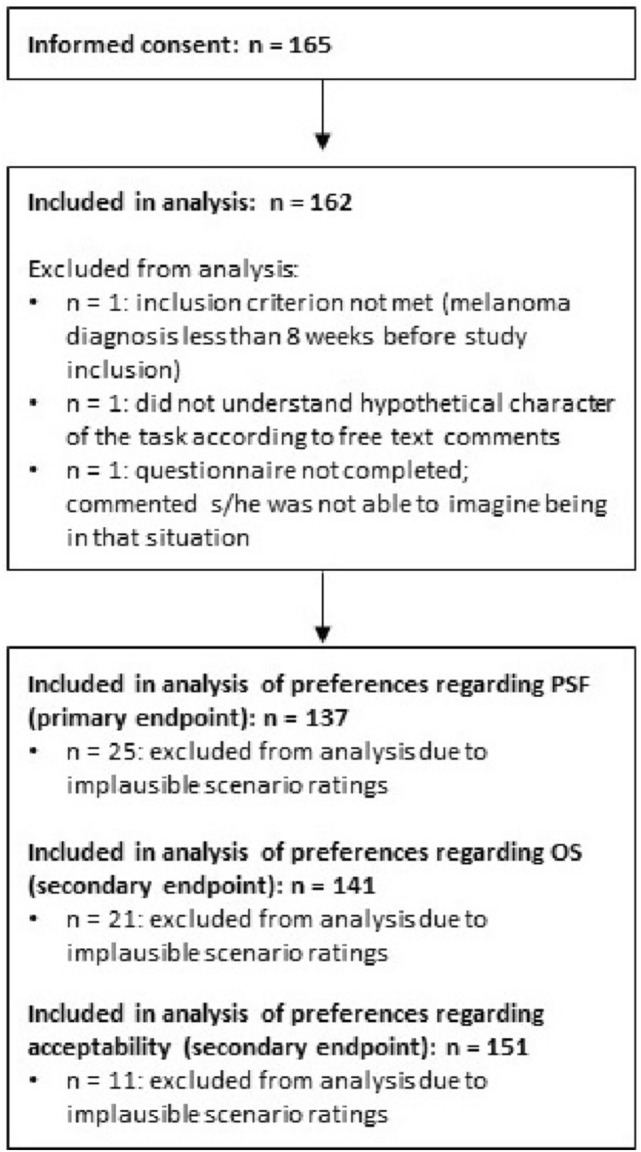
Study flowchart represented included, excluded, and analyzed participants

Out of 165 patients who gave informed consent, 3 had to be excluded from analysis for different reasons (Fig. 1). Regarding analysis of the scenario ratings, between 11 and 25 patients had to be excluded, with *n* = 137 analyzable for the primary endpoint.

### Patient characteristics

To characterize the study cohort, socio-demographics of the full analysis set of 162 patients are presented (Tables 1, 2).

**Table 1:** Age, BMI (body mass index), years since melanoma diagnosis, job (*n* = 162)

	Valid	Missing	Mean	Median	SD	Range
Age in years	160	2	60.2	60.0	13.6	24.0–93.0
BMI	159	3	26.7	25.9	4.7	17.3–44.1
Years since melanoma diagnosis	145	17	3.3	1.0	5.0	0.0–32.0
Job hours per week (only employed patients, *n* = 84)	77	7	35.5	39.0	9.8	7.0–60.0

**Table 2:** Sex, family, living, income, nationality, school, education, job (*n* = 162)

	Frequency	Percent
Sex		
Female	86	53.1
Male	74	45.7
Number missings	2	2.1
Family		
Single	10	6.2
Committed relationship	13	8.0
Married	117	72.2
Living apart	2	1.2
Divorced	8	4.9
Widowed	9	5.6
Committed relationship and divorced	1	0.6
Number missing	2	2.1
Living		
Alone	21	13.0
With 1 person	84	51.9
With 2 persons	28	17.3
With 3 persons	17	10.5
With 4 persons	4	2.5
With 5 persons	1	0.6
Not alone, but without number of persons stated	4	2.5
Number missing	3	1.9
Nationality		
German	142	87.7
Swiss	12	7.4
Czech and Swiss	1	0.6
Danish	1	0.6
Greek	1	0.6
Dutch	1	0.6
Number missing	3	1.9
School		
Secondary school certificate (9 years) [Hauptschulabschluss]	27	16.7
Secondary school certificate (10 years) [Realschulabschluss]	44	27.2
Advanced technical college certificate [Fachhochschulreife]	22	13.6
A-Levels [Abitur]	63	38.9
Other	3	1.9
Number missing	3	1.9
Education		
Completed apprenticeship	104	64.2
University degree	40	24.7
Advanced technical college degree	33	20.4
None of the above	2	1.2
Number missing	2	1.2
Job		
Employed	84	51.9
Leave	1	0.6
Retired	70	43.2
Early retired (because of melanoma)	2	1.2
Housewife/houseman	19	12.7
Student	2	1.2
Unemployed	0	0.0
Voluntary work	2	1.2
Number missing	2	1.2

The patient cohort (95% were German/Swiss, 5% had different nationalities) nearly equally consisted of female (57%) and male (47%) subjects. Patients were between 24 and 93 years of age (median 60 years) and had received the melanoma diagnosis in median 1 year ago (SD 5 years, range 0–32 years). Most were married and living with one person. The majority was employed with a median of 39 h/week, or retired. 4% reported that they were currently affected by another cancer and further 14% named other antecedent malignancies (Table 3).

**Table 3:** Patients’ self-experiences with cancer (*n* = 162)

	Frequency	Percent
Self-affected by cancer		
Yes, currently	6	3.7
Basal cell carcinoma	1	0.6
Desmoid abdomen	1	0.6
Follicular lymphoma	1	0.6
Lung cancer	1	0.6
Non-melanoma skin cancer	1	0.6
Malignant melanoma	1	0.6
Yes, in the past	22	13.6
Acoustic neuroma	1	0.6
Basalioma	1	0.6
Basalioma (nose)	1	0.6
Leukemia	1	1.2
Cervical cancer	3	1.8
Hair cell leukemia	1	0.6
Mamma carcinoma	4	2.5
Kidney and prostate	1	0.6
Non-Hodgkin lymphoma (nose) and prostate	1	0.6
Prostate	4	2.5
Vulvar carcinoma	1	0.6
Non-Melanoma skin cancer	2	1.2
Not specified	1	0.6
No	133	82.1
Number missing	2	1.2
Persons close to participant affected by cancer		
Yes, close relatives	101	62.3
Yes, partner	20	12.3
Yes, close friends	35	21.6
Yes, others	13	8.0
Work colleagues and acquaintances	5	3.1
Uncles/ aunts	4	2.5
Nieces/ nephews	1	0.6
Parents in law	2	1.2
Stepchildren	1	0.6
No	27	16.7
Number missing	2	1.2

The majority of patients had closely related persons affected by cancer which were relatives in 62%, friends in 21% and life partners in 12%. Most frequent concurrent comorbidities reported by patients were arterial hypertension (40%), thyroidal diseases (15%) and lipometabolic disorders (14%).

### Scenario rating with regard to disease-free survival

The primary endpoint, the patient preferences during TT with severe side effects in an adjuvant treatment setting, defined as the minimally required benefit in terms of the additional chance of 5-year DFS, was evaluated in 132 patients. Patients required in median a reduction of relapses by 35 out of 70 (mean 37.5, SD 22.0, range 0–70, 95% confidence interval 33.7–41.3).

For all scenarios, patients on average required a high number of prevented relapses by the respective treatment in order to accept it (Tables 4, 5).

**Table 4:** Scenario rating regarding DFS: Minimal number of patients out of 70 with prevented relapse that is needed for the treatment to be accepted (*n* = 137)

	Valid	Missing	Mean	Median	SD	Range	95% confidence interval
Scenario 1	132	5	21.1	10.0	21.4	0–70	17.4–24.7
Scenario 2	130	7	27.9	30.0	22.0	0–70	24.1–31.7
Scenario 3	132	5	37.5	35.0	22.0	0–70	33.7–41.3
Scenario 4	133	4	18.6	10.0	19.8	0–70	15.2–22.0
Scenario 5	132	5	24.2	20.0	20.3	0–70	20.7–27.7
Scenario 6	132	5	29.2	27.5	22.2	0–70	25.4–33.0
Scenario 7	132	5	42.4	50.0	20.9	0–70	38.8–46.0
Scenario 8	134	3	21.6	15.0	20.3	0–70	18.1–25.1
Scenario 9	134	3	27.1	29.0	20.9	0–70	23.6–30.7
Scenario 10	133	4	31.1	35.0	21.4	0–70	27.5–34.8
Scenario 11	131	6	43.0	50.0	21.3	0–70	39.3–46.7

**Table 5:** *P* values of the paired *t* tests on differences in mean scenario ratings regarding DFS

Scenario	1	2	3	4	5	6	7	8	9	10
Scenario 1	–									
Scenario 2	** < 0.001**	–								
Scenario 3	** < 0.001**	** < 0.001**	–							
Scenario 4	**0.005**	** < 0.001**	** < 0.001**	–						
Scenario 5	** < 0.001**	** < 0.001**	** < 0.001**	** < 0.001**	–					
Scenario 6	** < 0.001**	0.063	** < 0.001**	** < 0.001**	** < 0.001**	–				
Scenario 7	** < 0.001**	** < 0.001**	** < 0.001**	** < 0.001**	** < 0.001**	** < 0.001**	–			
Scenario 8	0.500	** < 0.001**	** < 0.001**	** < 0.001**	** < 0.001**	** < 0.001**	** < 0.001**	–		
Scenario 9	** < 0.001**	0.640	** < 0.001**	** < 0.001**	**0.003**	**0.014**	** < 0.001**	** < 0.001**	–	
Scenario 10	** < 0.001**	**0.003**	** < 0.001**	** < 0.001**	** < 0.001**	**0.019**	** < 0.001**	** < 0.001**	** < 0.001**	–
Scenario 11	** < 0.001**	** < 0.001**	** < 0.001**	** < 0.001**	** < 0.001**	** < 0.001**	0.426	** < 0.001**	** < 0.001**	** < 0.001**

Bold numbers indicate significant pairwise differences (*p* < 0.05)

In case of (a) no or (b) mild-to-moderate side effects patients requested similar reduction of relapses (10 and 30 for TT, 10 and 20 for ICI, 15 and 29 for cICI; median). Most ratings of scenarios were statistically different from each other (Table 5). Acceptance decreased with severity of side effects. For TT with severe side effects, a median of 35 avoided relapses was required. In contrast, ICI or cICI were expected to reduce the number of relapses by 50 in case of severe side effects, each.

### Scenario rating with regards to overall survival

For all scenarios, patients on average required a high number of prevented deaths by the respective treatment in order to accept it (Tables 6, 7).

**Table 6:** Scenario rating regarding OS: minimal number of patients out of 50 with prevented death that is needed for the treatment to be accepted (*n* = 141)

	Valid	Missing	Mean	Median	SD	Range	95% Confidence interval
Scenario 1	136	5	14.2	5	15.1	0–50	11.6–16.7
Scenario 2	134	7	18.1	15	16.0	0–50	15.3–20.8
Scenario 3	136	5	25.2	25	16.6	0–50	22.4–28.0
Scenario 4	137	4	13.5	5	15.0	0–50	11.0–16.1
Scenario 5	136	5	17.3	15	15.2	0–50	14.7–19.8
Scenario 6	136	5	20.2	20	16.5	0–50	17.4–23.0
Scenario 7	135	6	30.1	30	15.8	0–50	27.4–32.8
Scenario 8	138	3	15.1	10	15.0	0–50	12.6–17.6
Scenario 9	138	3	18.8	20	15.3	0–50	16.2–21.4
Scenario 10	137	4	21.7	20	16.2	0–50	18.9–24.4
Scenario 11	134	7	30.7	35	16.1	0–50	28.0–33.5

**Table 7:** *P* values of the paired *t* tests on differences in mean scenario ratings regarding OS (*n* = 141)

Scenarios	1	2	3	4	5	6	7	8	9	10
Scenario 1	–									
Scenario 2	** < 0.001**	–								
Scenario 3	** < 0.001**	** < 0.001**	–							
Scenario 4	0.332	** < 0.001**	** < 0.001**	–						
Scenario 5	** < 0.001**	0.559	** < 0.001**	** < 0.001**	–					
Scenario 6	** < 0.001**	** < 0.001**	** < 0.001**	** < 0.001**	** < 0.001**	–				
Scenario 7	** < 0.001**	** < 0.001**	** < 0.001**	** < 0.001**	** < 0.001**	** < 0.001**	–			
Scenario 8	0.104	** < 0.001**	** < 0.001**	**0.002**	** < 0.001**	** < 0.001**	** < 0.001**	–		
Scenario 9	** < 0.001**	0.187	** < 0.001**	** < 0.001**	0.062	**0.014**	** < 0.001**	** < 0.001**	–	
Scenario 10	** < 0.001**	** < 0.001**	** < 0.001**	** < 0.001**	** < 0.001**	**0.025**	** < 0.001**	** < 0.001**	** < 0.001**	–
Scenario 11	** < 0.001**	** < 0.001**	** < 0.001**	** < 0.001**	** < 0.001**	** < 0.001**	0.338	** < 0.001**	** < 0.001**	** < 0.001**

Bold numbers indicate significant pairwise differences (*p* < 0.05)

In case of (a) no or (b) mild-to-moderate side effects patients requested a similar reduction of deaths (5 and 15 for TT, 5 and 15 for ICI, 10 and 20 for cICI; median). Most ratings of scenarios were statistically different from each other (Table 7). Acceptance decreased with severity of side effects: for TT with severe side effects, a median of 25 avoided deaths was required, in contrast to 30 avoided deaths for ICI or 35 deaths in case of cICI (*p* < 0.001).

### Scenario rating: acceptability

As shown in Tables 8, 9, acceptability ratings as measured with VAS (scale 0–100) were statistically different for most scenario pairs.

**Table 8:** Scenario rating regarding acceptability: Judgement of the scenarios on a VAS from 0 to 100 (*n* = 151)

	Valid	Missing	Mean	Median	SD	Range	95% Confidence interval
Scenario 1	151	0	92.4	100.0	13.2	20–100	90.2–94.5
Scenario 2	150	1	70.3	73.0	20.3	0–100	67.1–73.6
Scenario 3	149	2	45.5	50.0	23.0	0–100	41.7–49.2
Scenario 4	149	2	93.7	100.0	12.0	2–100	91.8–95.7
Scenario 5	150	1	77.0	80.0	16.8	30–100	74.3–79.7
Scenario 6	150	1	65.2	70.0	22.3	0–100	61.6–68.8
Scenario 7	150	1	35.3	34.0	21.0	0–100	31.9–38.7
Scenario 8	150	1	88.5	90.0	14.4	30–100	86.2–90.8
Scenario 9	149	2	74.3	80.0	17.0	20–100	71.5–77.0
Scenario 10	150	1	64.0	65.0	20.8	0–100	60.6–67.3
Scenario 11	150	1	35.5	30.5	22.2	0–100	31.9–39.1
Scenario 12	150	1	22.6	10.0	24.4	0–100	18.6–26.5

**Table 9:** *P* values of the paired *t* tests on differences in mean scenario rating (*n* = 151)

	1	2	3	4	5	6	7	8	9	10	11
Scenario 1	–										
Scenario 2	** < 0.001**	–									
Scenario 3	** < 0.001**	** < 0.001**	–								
Scenario 4	0.109	** < 0.001**	** < 0.001**	–							
Scenario 5	** < 0.001**	** < 0.001**	** < 0.001**	** < 0.001**	–						
Scenario 6	** < 0.001**	**0.001**	** < 0.001**	** < 0.001**	** < 0.001**	–					
Scenario 7	** < 0.001**	** < 0.001**	** < 0.001**	** < 0.001**	** < 0.001**	** < 0.001**	–				
Scenario 8	**0.002**	** < 0.001**	** < 0.001**	** < 0.001**	** < 0.001**	** < 0.001**	** < 0.001**	–			
Scenario 9	** < 0.001**	**0.002**	** < 0.001**	** < 0.001**	**0.005**	** < 0.001**	** < 0.001**	** < 0.001**	–		
Scenario 10	** < 0.001**	** < 0.001**	** < 0.001**	** < 0.001**	** < 0.001**	0.261	** < 0.001**	** < 0.001**	** < 0.001**	–	
Scenario 11	** < 0.001**	** < 0.001**	** < 0.001**	** < 0.001**	** < 0.001**	** < 0.001**	0.824	** < 0.001**	** < 0.001**	** < 0.001**	–
Scenario 12	** < 0.001**	** < 0.001**	** < 0.001**	** < 0.001**	** < 0.001**	** < 0.001**	** < 0.001**	** < 0.001**	** < 0.001**	** < 0.001**	** < 0.001**

Bold numbers indicate significant pairwise differences (*p* < 0.05)

Most scenarios ware rated as completely unacceptable (VAS = 0) by less than 1% of the participants. Exceptions were scenario 3, 7, and 11 (severe side effects; all rated as unacceptable by < 5% of patients) and scenario 12 (melanoma relapse, rated as unacceptable by 17% of patients). Thus, there was a marked difference between scenario 12 and the remaining scenarios (Table 9). The acceptance of a treatment option without side effects was high (median of 100/100/90 for TT; ICI; (c)ICI); in case of mild-to-moderate side effects, this acceptance dropped (median of 83/70/60 for TT; ICI; (c)ICI) and was lowest in case of severe side effects (median of 50/34/31 for TT; ICI; (c)ICI). Melanoma relapse was rated worst with a median of 10 (range 19–27).

### Scenario rating by socio-economic characteristics

In most scenarios, there was a low correlation between DFS ratings and age (*r* < 0.3) in that older patients tended to require higher effectiveness in order to accept a treatment (data not shown). Gender, income, or co-morbidities did not show any association with DFS or OS rating. Neither did scenario ratings correlate with years since melanoma diagnosis. Average scenario ratings regarding acceptability, DFS or OS also did not differ between patients with versus without experience with cancer, except for scenarios 8 and 9 (cICI without vs, with mild to moderate side effects).

### Dosage form preferences: infusion vs. oral medication

Most patients stated it was okay for them to take the medicine on their own (63% “totally agree”; Table 10).

**Table 10:** Absolute and total frequencies for medication preferences (*n* = 162)

	Frequency	Percent of valid responses	Cumulative %
Self-applied medication			
Totally agree	100	63.3	63.3
Rather agree	25	15.8	79.1
Undecided	21	13.3	92.4
Rather disagree	12	7.6	100.0
Totally disagree	0	0.0	0.0
Missing	4	–	–
Supervised medication			
Totally agree	20	12.7	12.7
Rather agree	33	20.9	33.5
Undecided	33	20.9	54.4
Rather disagree	55	34.8	89.2
Totally disagree	17	10.8	100.0
Missing	4	–	–
Rather visits than self-application			
Totally agree	17	10.6	10.6
Rather agree	33	20.6	31.3
Undecided	27	16.9	48.1
Rather disagree	41	25.6	73.8
Totally disagree	42	26.3	100.0
Missing	2	–	–
Acceptance of long doctor’s appointments			
Totally agree	36	22.5	22.5
Rather agree	46	28.7	51.2
Undecided	29	18.1	69.4
Rather disagree	39	24.4	93.8
Totally disagree	10	6.3	100.0
Missing	2	–	–
Compliance to a strict intake schedule			
Totally agree	85	53.5	53.5
Rather agree	42	26.4	79.9
Undecided	21	13.2	93.1
Rather disagree	9	5.7	98.7
Totally disagree	2	1.3	100.0
Missing	3	–	–
Importance of treatment method (infusion vs. pill)			
Totally agree	64	40.3	40.3
Rather agree	38	23.9	64.2
Undecided	39	24.5	88.7
Rather disagree	15	9.4	98.1
Totally disagree	3	1.9	100.0
Missing	3	–	–
Do you currently use medication on a regular basis?			
No	61	37.9	37.9
Yes	100	62.1	100.0
Missing	1	–	–
Is there somebody who can accompany you to the infusions?			
No	29	18.6	18.6
Yes	127	81.4	100.0
Missing	6	–	–

Patients tended not to see benefits in supervised medication. On average, patients rather disagreed that they would accept infusions and doctor visits and tended to state they would accept appointments that take several hours. Patients declared that they could stick to a precise intake schedule and that it the administration method (infusion or pills) was important to them. On the horizontal VAS, most patients indicated a preference for pills with a median of 31 on the scale from − 100 (infusion preferred) to + 100 (pills preferred) (mean 26.1, SD 61.6, range − 100 to 100, *n* = 161).

### EQ-5D-5L and EQ VAS

Generic HRQoL as measured with the EQ-5D-5L was high with a mean of 0.94 on the scale of 0–1 (median 0.97; SD 0.09). Self-rated health as measured with the EQ VAS was also high with a mean of 80.9 (SD 14.8) on the scale of 0–100. Neither value is lower than in the German adult population (EQ-5D-5L: 0.88; EQ VAS: 71.6) (Grochtdreis et al. 2019).

HRQoL mostly did not correlate with scenario ratings, except for DFS ratings of scenarios 8, 9 and 10 on (c)ICI treatment (patients with better quality of life accepted combination treatment already with a lower number of relapses, but with a small effect size of *r* = − 0.18). Self-rated health (EQ VAS) mostly did not correlate with scenario ratings, except for VAS ratings of scenarios 1 and 4 (no side effects during TT or ICI: patients with better self-rated health rated assessed adjuvant treatment without side effects more positively, again with small effect sizes of *r* = 0.25 and 0.17, respectively).

### HADS-D

Anxiety and depression ratings were low with an average of 4.6 (anxiety, median 4.0, SD 3.0) and 2.7 (depression, median 2.0, SD 2.6) on the 0–21 scale, which was not higher than in the general German population (Hinz and Brahler 2011). Anxiety did not correlate with scenario ratings regarding DFS or OS, but regarding acceptability of scenarios as measured with the VAS: here, scenarios 1–3, 7, 10, and 11 were rated less acceptable by patients with higher anxiety, albeit with a small effect size of *r* < 0.2.

Depression correlated significantly with the majority of scenario ratings regarding DFS, OS and acceptability (7 out of 11 scenarios each) in that patients with higher depression tended to require higher effectiveness for a treatment to be acceptable, and rated the scenarios less acceptable; again, effect sizes were small (*r* < 0.32).

### EORTC QLQ-C30

Cancer-related quality of life was mostly good with a mean overall score of 87.1 (median 89.4, SD 11.2) on the 0–100 scale. Descriptively, these values are even higher than in the German general population (Waldmann et al. 2013). Cancer-related quality of life mostly did not correlate with scenario ratings, except for the VAS rating of scenario 3 (patients with lower HRQoL rated TT with severe side effects more negatively, *p* = 0.019).

## Discussion

This study elicited the acceptance of treatment related toxicity versus reduction of recurrence or death in German and Swiss melanoma patients in the setting of adjuvant therapy. As all current treatment approaches induce positive effects on DFS and DMFS, treatment decision for or against a treatment modality is very often driven by the risk for toxicity. Our analysis highlights the differences in patients’ preferences between current adjuvant treatment options in melanoma. For the former adjuvant standard treatment with interferon alpha-2a and 2b, we evaluated in a similar approach the patient preferences towards toxicity in that adjuvant setting in a multicenter approach in German skin cancer centers (the “DeCOG GERMELATOX survey) (Kaehler et al. 2016). In that analysis, we found that patients rated melanoma relapse worse compared to even severe treatment related side effects. Therefore, we intended to address the same questions of patients' acceptance regarding current adjuvant treatment approaches in melanoma.

### Association with patient characteristics

In our study, DFS ratings correlated significantly with age, older patients tended to require higher effectiveness in order to accept an adjuvant treatment; however, effect sizes were small. These results are similar to our previous GERMELATOX analysis that evaluated the patient preferences with regard to adjuvant IFN (Kaehler et al. 2016). In contrast, Weilandt et al. showed in a discrete choice approach in melanoma patients that with increasing age toxicity and impact on their daily routine outbalanced efficacy (Weilandt et al. 2021). They also found that married patients and patients with a higher level of education have higher expectations towards treatment efficacy (Weilandt et al. 2021).

In our study, average scenario ratings regarding acceptability or DFS did not differ between patients previously diagnosed with cancer and those without. Average scenario ratings regarding OS did not differ between patients with experience with cancer and those without, except for scenarios 8 and 9 (cICI without or only mild to moderate side effects) but, again, with small effect sizes only.

The wide range of ratings in our study demonstrates how different patients’ perspectives are.

Additionally, scenario ratings did not correlate with years since melanoma diagnosis. The hypothesis that previous cancer experience could affect treatment associated outcome ratings has also been evaluated by Weiss et al. who found that patients and also physicians previously affected by cancer valued life prolongation by melanoma treatment more positively as compared to healthy controls or physicians without personal cancer experience, respectively (Weiss et al. 2020).

### Difference between perception of TT versus (c)ICI

The differences between TT and (c)ICI with regard to the specific side effect spectrum were reflected in different perspectives of patients towards the acceptability of these drugs. Interestingly, patients were more willing to accept TT-induced severe side effects (scenario 3) compared to severe side effects induced by (c)ICI (scenarios 7 and 11). Patients rated potentially lethal or not resolving side effects worse. Most of the scenarios with exception of scenarios 3, 7, and 11 (severe side effects; all < 5%) and scenario 12 (melanoma relapse, 17%) were rated as completely unacceptable (VAS = 0) by less than 1% of the patients, showing the immense willingness of German and Swiss patients to tolerate treatment-related side effects. Interestingly, a study by Mansfield et al. showed that patients were more willing to accept TT-associated pyrexia if the drug efficacy and, therefore, their outcome benefit is known (Mansfield et al. 2021). Patients in the adjuvant setting were more willing to accept pyrexia compared to patients with advanced melanoma (Mansfield et al. 2021). This demonstrates that it is crucial to explain thoroughly the potential treatment benefit in order to achieve the optimal patient willingness to accept adjuvant treatment of melanoma.

The more negative perception of severe side effects during adjuvant treatment with (c)ICI compared to TT has also been confirmed by the comparison of the acceptability of scenarios. This can be explained by the possibility of long-lasting toxicity with sequelae and as well potentially fatal course of autoimmune side effects (Wang et al. 2018). In contrast, melanoma patients evaluated by Stehnejem et al. and also Weilandt et al. rated skin toxicity in terms of rash much more negatively than organ specific autoimmune side effects like e.g. colitis (Stenehjem et al. 2019; Weilandt et al. 2021). A possible explanation would be the visibility of rash, its disfiguring potential as well as the impact of itch on quality of life. In contrast, autoimmune side effects need to be managed by systemic steroids, but are mostly invisible for the social sphere of the patients. Finally, any differences between our findings with previous studies can stem from different preference elicitation methods used.

The difference in the mode of administration between c(ICI) and TT might also be a reason for melanoma patients to rate TT more positively. Our patients preferred the autonomy of an oral medication whereas the melanoma cohort of Weilandt et al. (AJCC stage IIC-IV, disease free or with tumor manifestations) favored infusions significantly (Weilandt et al. 2021). This might be explained by the fact that in our patient cohort, the decision for melanoma treatment and treatment regimen was an entirely fictitious scenario. Therefore, they might value the autonomy of an oral medication higher, whereas patients facing the adjuvant treatment decision in a real scenario perhaps are somewhat overwhelmed by the challenge to understand the process and, therefore, prefer to delegate the treatment responsibility regarding medication intake to their physician (Mansfield et al. 2021).

### Association with psychological aspects

Overall, our patients showed a good psychological status in terms of generic and cancer-related HRQoL as well as self-rated health. Cancer-related HRQoL mostly did not correlate with scenario ratings, except for the VAS rating of scenario 3 (TT with severe side effects), in which patients with a worse HRQoL tended not to accept severe TT-induced side effects. Health-related HRQoL mostly did not correlate with scenario ratings, except for DFS ratings of scenarios 8, 9 and 10 on (c)ICI treatment (patients with better quality of life being were more willing to accept combination treatment with a lower number of relapses is prevented). Self-rated health (EQ VAS) mostly did not correlate with scenario ratings, except for VAS ratings of scenarios 1 and 4 (no side effects during TT or ICI): patients with better health rated a treatment setting without side effects very positively. However, all these effects were only small and thus may not be of clinical relevance.

### Do current treatment options meet our expectations?

The most relevant question is if current treatment options meet our patients' expectations. For DFS, patients' expectations towards efficacy differed between the three treatment modalities only in a range of 6 percentage points despite the distinct rate of grade 3–4 adverse events (range 14.4–71.0%) (Table 11).

**Table 11:** Comparison between patient preferences in this study and efficacy demonstrated in clinical trials: DFS

5y-DFS (%)	Expected efficacy (all grades of tolerability)	Expected efficacy (in case of severe side effects)	Grade 3–4 side effects in clinical trials	Efficacy in clinical trials
TT	55	65	41	52 (Dummer et al. 2020)
ICI	57	68	14.4	47.1–66.4 (4 y, Larkin et al. 2022)
cICI	62	71	71	64.2 (4 y, Livingstone et al. 2022)

In general, the treatment efficacy found in clinical trials (Dummer et al. 2020; Larkin et al. 2022; Livingstone et al. 2022; Schadendorf et al. 2022) seems to be able to meet the expectations in terms of DFS found in our patient cohort; however, some follow-up data still is immature and for (c)ICI, only 4-year DFS data is available so far. In contrast, our patients expected higher DFS rates in case of severe side effects than found in clinical studies, so in this situation efficacy would not be high enough for the patients. For OS (Table 12), the results are similar, but in case of severe side effects, the gap between expectations and the efficacy demonstrated in clinical trials so far seems to be smaller in case of using cICI.

**Table 12:** Comparison between our patients ‘preferences and efficacy demonstrated in clinical trials: OS

5 y-OS (%)	Expected efficacy (all grades of tolerability)	Expected efficacy (in case of severe side effects)	Grade 3–4 side effects in clinical trials	Efficacy in clinical trials
TT	65	75	41	64.6–75.3 (4 y DMFS, Schadendorf et al. 2022)
ICI	68	80	14.4	55.7–70 (4 y DMFS, Larkin et al. 2022)
cICI	71	85	71	83.3 (4 y OS, Livingstone et al. 2022)

In contrast, the gap for TT and ICI is more pronounced, which implies that patients would not appreciate the risk–benefit ratio as much as for cICI. Of course, cICI is only used in selected patients in the adjuvant setting in AJCC stage IV.

### Limitations of the study

Limitations of our study included the selection of a patient population with only low-risk melanoma due to ethical reasons. Thus, the patients’ preferences were used as surrogates for those of patients in later disease stages. We did not analyze the perceptions of adjuvant melanoma treatment over time, so possible changes in the individual course of disease are not captured, but there is evidence that tumor stage does not necessarily influence patients' preferences (Livingstone et al. 2020). Therefore, our selection of melanoma patients might be less biased and thus particularly suitable to gain more information on patient preferences towards adjuvant treatment. Furthermore, our usual melanoma patient cohort consists of more male than female patients, while in our study, more female patients were willing to participate. Finally, patient preferences were elucidated based on hypothetical, yet realistic, scenarios, and are thus not completely comparable to real-life treatment decisions.

In conclusion, we determined in our German and Swiss melanoma patients a higher willingness to accept TT-induced side effects compared to severe side effects induced by (c)ICI. Compared with results from clinical trials, patient expectations towards efficacy and side effects are mostly met. In particular, patients treated with cICI and severe side effects would probably be able to achieve a survival improvement that is within the range needed for them to outweigh these toxicities.

Finally, German and Swiss patients rated melanoma recurrence and death much lower than the potential toxicity spectrum induced by TT or (c)ICI. Our results show a marked concordance to our previous Germelatox study (Liu et al. 2019) and describe our patients´ preferences for current melanoma treatment in the adjuvant setting. It is helpful for our clinical routine to have more detailed information on the individual preferences of our patients to improve balanced shared decision making.

## Supplementary Information

Below is the link to the electronic supplementary material.

Supplementary file1 (DOCX 884 KB)

## Data Availability

The datasets generated during and/or analysed during the current study are available from the corresponding author on reasonable request.
