# Supplementary material for: Preferences of German and Swiss melanoma patients for toxicities versus melanoma recurrence during adjuvant treatment (GERMELATOX-A-trial)
Source: J Cancer Res Clin Oncol. 2023 Jul 5;149(13):11705–18. doi: 10.1007/s00432-023-05027-z (PMC10465664; doi:10.1007/s00432-023-05027-z)
Supplement: Supplementary file 1 — Supplementary file1 (DOCX 884 KB) [file 432_2023_5027_MOESM1_ESM.docx]

**Scenario 1**

| **Adjuvant therapy:**  **Treatment WITHOUT side effects** | |
| --- | --- |
|  | You receive the medication combination „BRAF- and MEK-inhibitor“. This intervenes in the metabolism of the cancer cells, which fights any remaining cancer cells.  Please imagine the course of treatment described below. |
| Treatment | The medicines are taken as **tablets**. They should be taken at least one hour before or two hours after eating.  One tablet is taken **twice a day** at 12-hour intervals, the other additionally once a day.  The tablets should be taken at the same time each day.  The following **check-ups by a physician** are necessary during treatment:   - Ultrasound examination of the heart: monthly during the first 3 months and then at least every 3 months - ECG of the heart every 4 weeks |
| No side effects | Because the treatment has **no side effects**, …   - …you **do not** need any additional time to rest. - …you **do not** take any other medications. - … your usual sexual interest **remains** - … you **can** perform heavy work (e.g., moving large pieces of furniture or carrying heavy objects upstairs). - … you **can** perform strenuous recreational activities (e.g., play tennis or ride a bicycle at a moderate speed) - … you are **not** impaired in your ability to attend appointments (dates and the like)   You will receive this treatment for **one year**. |

| **Your evaluation of scenario 1** | |
| --- | --- |
| **Adjuvant therapy**  **Treatment WITHOUT side effects** | |
|  | Please try to imagine how you would feel in this situation. Under these conditions:  How would you rate scenario 1?  Please mark a point on the scale:  100%: absolutely bearable  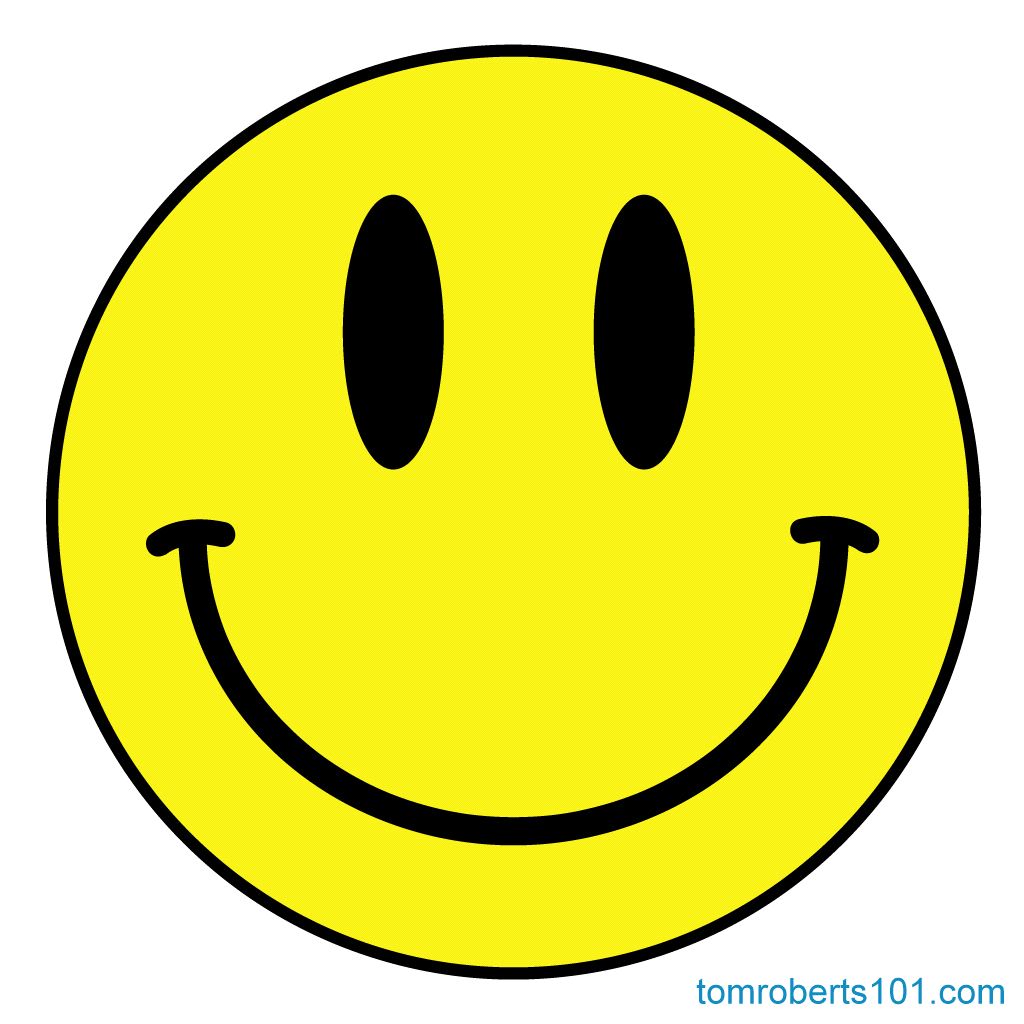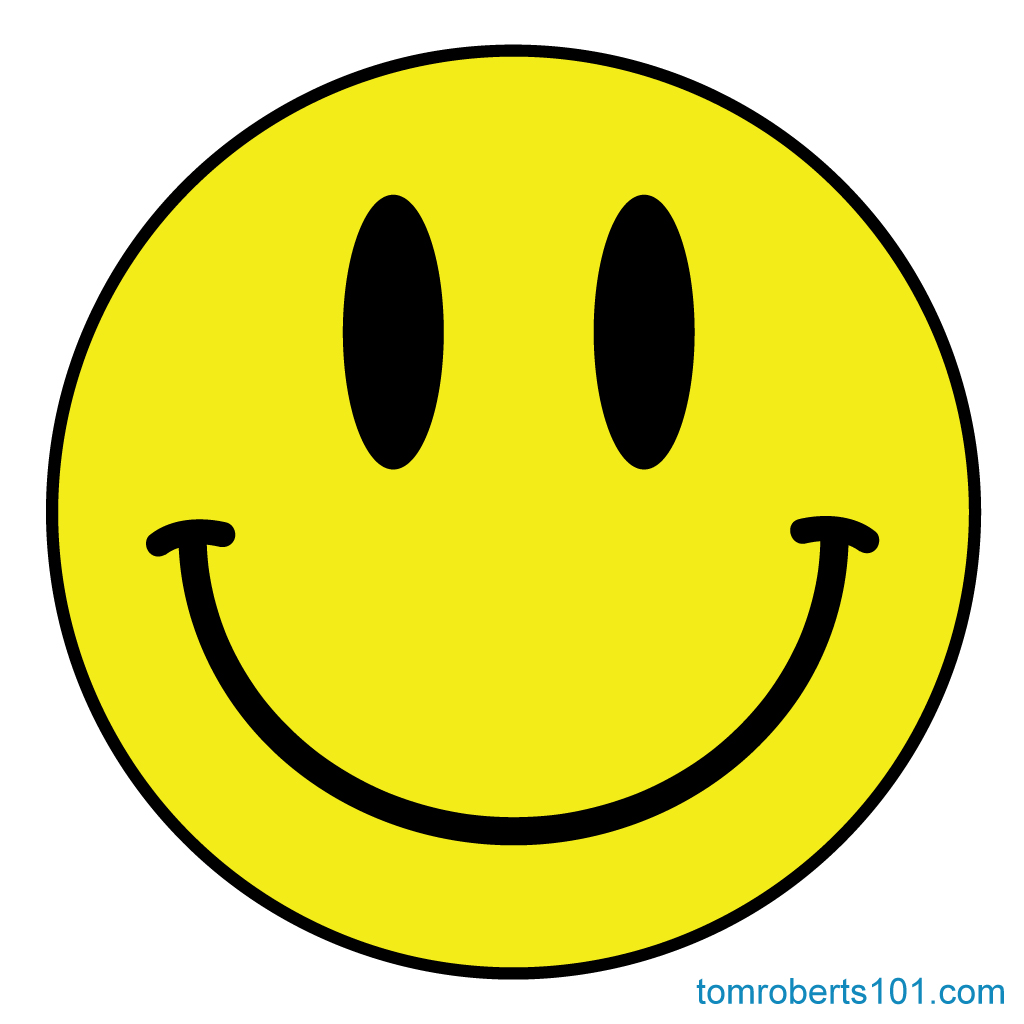  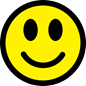  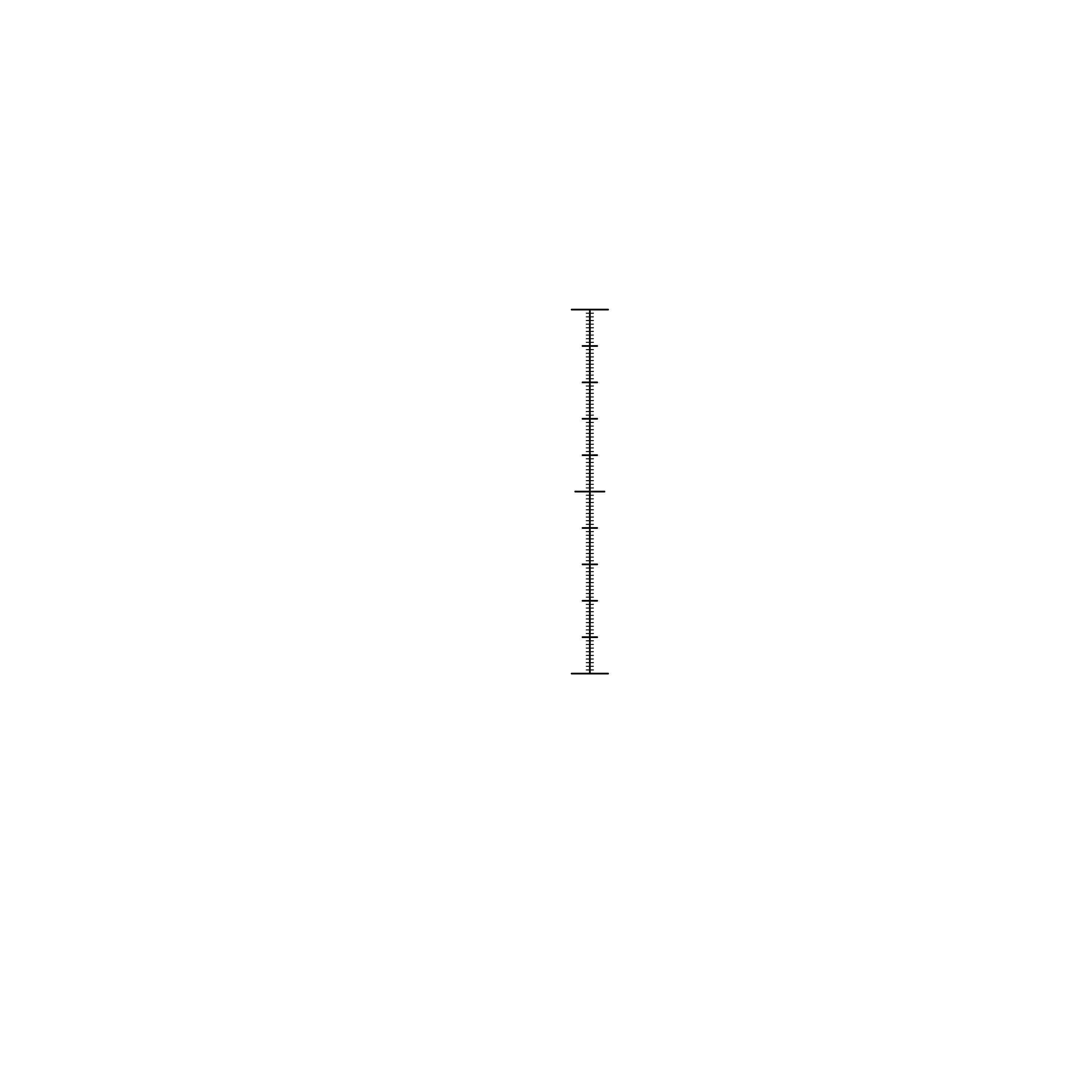  *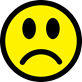*  0%:  absolutely intolerable |

|  | The treatment described in scenario 1 is called "supportive (adjuvant) treatment". It is intended to prevent a relapse, i.e., a progression of the cancer, after the end of the main treatment (e.g., surgery) and to increase the probability of survival.  **Without this supportive treatment**, patients with severe skin cancer have the following risk:   - **70 out of 100 patients have a relapse within 5 years after diagnosis.** If a relapse occurs, the skin cancer must be treated, for example, with surgery, radiation or further medication. - **50 out of 100 patients die within 5 years of diagnosis.**   Now please consider: Suppose there was only this one treatment to choose from. What would be the **minimum** effectiveness of the treatment described in scenario 1 ("**treatment WITHOUT side effects**") for you to choose it? In other words: How much would the treatment have to reduce the risk of relapse and death for it to be acceptable to you?  Without the treatment, **70 out of 100 patients** have a **relapse** within 5 years of diagnosis.  **I would accept the treatment described in scenario 1 if it prevented at least**  **_______________ of these 70 patients from relapsing.**  *(Note: The number you enter must be between 0 and 70.)*  Without the treatment, **50 out of 100 patients die** within 5 years of diagnosis.  **I would accept the treatment described in scenario 1 if it prevented at least**  **_______________ of these 70 patients from dying.**  *(Note: The number you enter must be between 0 and 50.)* |
| --- | --- |
